# The economic burden of diagnostic uncertainty on rare disease patients

**DOI:** 10.1186/s12913-024-11763-w

**Published:** 2024-11-12

**Authors:** Lukas Willmen, Lukas Völkel, Tina Willmen, Thilo Deckersbach, Siegfried Geyer, Annette Doris Wagner

**Affiliations:** 1 Department of Nephrology, Hannover Medical School, Hanover, Germany; 2 Clinic of Prosthetic Dentistry and Biomedical Materials Science, Hannover Medical School, Hanover, Germany; 3 Department of Psychology, DIPLOMA University, Bad Sooden-Allendorf, Germany; 4 Department of Medical Sociology, Hannover Medical School, Hanover, Germany

**Keywords:** Rare diseases, Health economics, Diagnostics, Diagnostic decision support system

## Abstract

**Background:**

It often takes a long time before a rare disease is diagnosed. Without a diagnosis, the right therapy often cannot be carried out and without the right therapy, the patients are denied the opportunity for a cure or relief from their symptoms.

In addition, rare diseases can also have economic consequences for those affected. This study aimed to investigate the extent to which a rare disease affects the income and work performance of the patients concerned and whether the use of AI in diagnostics would have the potential to reduce economic losses.

**Methods:**

The work performance and income of 71 patients of the outpatient clinic for rare inflammatory systemic diseases with renal involvement at Hannover Medical School were analyzed during the course of the disease. The WHO Health and Work Performance Questionnaire (HPQ) was used to collect data. During the patient interviews, the questionnaire was completed four times: at the onset of the first symptoms, when a diagnostic decision support system (DDSS) would have suggested the correct diagnosis, at the time of diagnosis and at the current status.

**Results:**

With the onset of the diagnostic odyssey, the monthly net income of the patients under study dropped by an average of 5.32% due to lower work performance or work absenteeism. With the correct diagnosis, the original or even a better income of 11.92% could be achieved. Loss of income due to illness was more massive in patients with a rare disease with joint, muscle and connective tissue involvement than in patients with rare vasculitides. If a DDSS had been used, the loss of income would have been 2.66% instead of the actual 5.32%.

**Conclusion:**

Rare diseases resulted in temporary or existing income losses in 28.17% of the patients. Losses in work performance and income were related to the type of disease and were more pronounced in patients with joint, muscle or connective tissue disease than in patients with rare vasculitides.

The use of a DDSS may have the potential to reduce the negative income effects of patients through earlier correct diagnosis.

**Trial registration:**

Retrospectively registered.

## Background

### Difficulties in diagnosing rare diseases

In Europe, a disease is considered rare if more than 5 in 10.000 people are affected by the disease. There are 30 million people in Europe alone who suffer from a rare disease. Worldwide, the number is even higher at 300 million [1].

Patients with rare diseases often go through a long period of uncertainty until they receive a definitive diagnosis. Affected patients wait an average of 4 to 5 years for a correct diagnosis [2–4]. Causes for the long diagnostic path can be the high number of rare diseases, their unknown nature [5], but also administrative inadequacies, such as long waiting times for medical appointments [6, 7] or lack of interdisciplinary exchange [8].

Until correct diagnosis patients are often misdiagnosed or wrongly treated [9, 10].

### The socioeconomic burden of patients with rare diseases

A rapid diagnosis is important not only for the treatment of the physical symptoms, but also for the psychosocial health of the patients, as patients with the disease often suffer from depression, anxiety and stress, which, in turn, can lead to social withdrawal [11]. The heavy physical and psychological burden of rare diseases also affects everyday working life, it reduces the ability to work which leads to unemployment and financial problems [11, 12]. This is also shown by Heuyer et al. in a barometer in which 51% of the respondents stated that they had not worked or had given up their job because of their rare disease [13]. A European survey with 1095 respondents reflects the major impact of the disease on working life as follows: 21% of the respondents reported to have been absent because of their disease for more than 90 days per year [14].

Chan et al. using Sjögren’s syndrome as an example, calculated the average annual productivity losses due to absenteeism and presenteeism at $41,094 [15].

Further factors that have an influence on the income are the lack of workplace adaptation for people with rare diseases as well as encountering discrimination because of their disease. A survey showed that 76% of the patients felt disadvantaged at work and 67% had not been promoted because of their disease [14].

### Advantages through the use of diagnostic decision support systems (DDSS)

The use of a diagnostic decision support system (DDSS) and also a rapid referral to expert centers may accelerate the diagnostic process and make earlier therapy possible [7, 16–18]. In the outpatient clinic for rare inflammatory systemic diseases with renal involvement at Hanover Medical School (MHH), Ada DX, the research prototype of a diagnostic decision support system, was evaluated for diagnostic support in an own study. Ada DX is a knowledge-based, probability-considering system designed to assist physicians in making diagnoses. The system is regularly updated by experts to reflect the latest scientific findings. In 2019, Ronicke et al. demonstrated in a retrospective study involving 93 patients with rare diseases that in 42 cases Ada DX would have suggested the correct diagnosis before the actual clinical diagnosis [17]. In 2021 T. Willmen et al. showed in another study with patients from this previously studied patient population that earlier diagnosis by Ada DX would have resulted in only 51–68% of total diagnostic costs. The system could also have avoided frequent follow-up examinations [7]. Rare diseases are a particularly suitable field for diagnostic systems because of the complexity and the heterogeneity of symptoms that often cannot be subsumed under the area of a single medical discipline.

### Aim of the study

The aim of this study was to demonstrate the impact of rare diseases on productivity and income.

The hypothesis was that a correct diagnosis would have a positive effect on income.

A further hypothesis was that rare diseases with joint, muscle, or connective tissue involvement have a greater negative impact on productivity and income than rare diseases of small or large vessel vasculitides.

It was also analyzed what influence an earlier diagnosis through the DDSS would have had on the income development of the patients.

## Methods

### Setting

The data for this study were collected at the outpatient clinic for rare inflammatory systemic diseases with renal involvement at Hannover Medical School. MHH is one of 37 centers of expertise for rare diseases in Germany [19]. The project this paper is based on is carried out by an interdisciplinary team of scientists. Everyone contributes from the perspective of their own discipline. The head of the outpatient clinic for rare inflammatory systemic diseases is a specialist in both nephrology and rheumatology and has a profound and long-standing experience in the diagnosis and treatment of rare diseases [16]. The economic part is covered by an economist with profound expertise in the area of health economics. The other members of the group come from the fields of medicine, epidemiology and social sciences.

### Case selection

Only patients who had been included in the previous studies by Ronicke et al. [17] and T. Willmen et al. [7] were selected for the study.

In these patient cases, the course of the disease from the onset of symptoms to diagnosis was completely documented. Patients with long diagnostic odysseys were preferred [17]. In addition, patients had had a definite diagnosis for at least 2 years, which was also confirmed as correct during subsequent controls, which took place 3 to 4 times per year [16].

### The operating principle of Ada DX and the previous study

Symptoms and test results of patients are can be entered into the Ada DX diagnostic decision support system. Based on the results, the system creates two lists.

In addition to a probability list, that assigns suitable diagnostic suggestions to patient’s symptoms taking into account the probability of the presence of a disease, the system also generates a so-called fit list. The fit list does not take into account the probability of the presence of a disease, but is based on matching the entered symptoms. By creating the fit list, Ada DX is particularly well suited for diagnosing rare diseases [17].

In the previous retrospective study, Ronicke et al. extracted all diagnostic tests from patient records from the onset of symptoms and entered them into Ada DX in chronological order [17]. The time at which Ada DX suggested the correct diagnosis among the top five suggestions and the time at which Ada DX suggested the correct diagnosis in the first place were recorded [17].

These time points were used to analyze the financial impact that an earlier diagnosis by DDSS would have had for this study.

### Data collection

The World Health Organization’s Health and Work Performance Questionnaire (HPQ) [20, 21] was used for collecting the data. It measures the influence of the patients’ health status on their work performance in the form of presenteeism and absenteeism. The questionnaire was completed in person during the on-site interviews or by telephone.

Each patient interview comprised four rounds of questions at the following times in the course of the patient’s disease:

#### Time point 1 - first onset of symptoms

Time at which, according to patient records, the first doctor’s visit with disease-related symptoms occurred.

#### Time point 2 - top 1 fit suggestion in ADA DX

Time at which the diagnosis decision support system hypothetically presents the correct diagnosis in the first position after input of symptoms and examination results.

#### Time point 3 - definitive diagnosis by the expert

Time at which the correct diagnosis was actually given.

#### Time point 4 – under therapy

Time under therapy with correct diagnosis at time of interview.

Time points 1 and 3 were accurately dated in the available patient records and could be extracted from the records. Time point 2 at which the DDSS presented the correct diagnosis as top 1 suggestion was taken from the study by Ronicke et al. [17].

### Health and work performance questionnaire

The Health and Work Performance Questionnaire (HPQ) [20, 21] is a standardized questionnaire on health and work performance, which has already been used in many studies to quantify the costs of health problems at work [22]. For this study it was used in the 28-day version in order to consider the respective survey month. Instead of the usual income data in $, we used an equivalent salary query in € for our study.

First the demographic data were collected from all patients.

The first part of the questionnaire then asked about the patients’ work situation. In two further parts, questions were asked to determine absenteeism and presenteeism. Absenteeism describes the absence of employees from workplace, while presenteeism describes the quality of work when employees work despite an illness, although they could take a sick leave.

To assess absenteeism, the questionnaire included the following questions: *“How many hours does your employer expect you to work in a typical 7-day week? (*B4**; ***if it varies*,* estimate the average. If more than 97*,* enter 97.)”* Additionally, *“About how many hours altogether did you work in the past 4 weeks (28 days;*B6*)?”* The calculation for relative absenteeism was determined according to the scoring document using the formula [23]:

$$\:\frac{(4\times\:B4-B6)}{(4\times\:B4)}$$

For presenteeism, the questionnaire posed the following questions: *“On a scale from 0 to 10 where 0 is the worst job performance anyone could have at your job and 10 is the performance of a top worker*,* how would you rate the usual performance of most workers in a job similar to yours?”* (**B9**). Following this respondents were asked to rate their overall job performance on the days they worked during the past 4 weeks using the same scale (**B11)**. The calculation for relative presenteeism was also derived according to the scoring document using the formula [23]:

$$\:\frac{B11}{B9}$$

Relative absenteeism and relative presenteeism can be represented through a combined score. This score is calculated by determining the relative hours of work, defined as one minus relative absenteeism. The final score is then expressed as follows [23]:

$$\begin{aligned} \:Combined\:Score\:=&\:Relative\:Hours\:of\:Work\:\\&\times\:\:Relative\:Presenteeism\end{aligned}$$

Multiplying the value of the combined score by the net income for the month resulted in a monetary value of the score for the calculated time.

### Analysis of the income and productivity development of all disease courses

As income was a crucial element for the validity of our findings, it was assessed by asking patients directly during the interview. They were asked to provide income-related documents in order to minimize errors in the data and thus in our estimations.

For the analysis of income and productivity development, the mean values of all patient incomes and combined values at time points 1 (onset of symptoms), 3 (expert diagnosis) and 4 (under therapy) were calculated and presented. For a more detailed analysis, it was recorded how many patients showed an improvement, a worsening or a stagnation at the respective time points 3 (expert diagnosis) and 4 (under therapy).

Patients with a deterioration or improvement in income and productivity were also assessed and presented separately.

In order to capture the effects of early retirement, we determined how many patients retired earlier and, if so, at what average age. In addition, the effect on income of early retirement was calculated.

### Comparison of income and productivity development depending on the type of disease

Since the adverse effects on patients and the economic effects depend on the type of disease, we compared rare diseases with small or large vessel vasculitides and rare diseases with joint, muscle, or connective tissue involvement, and divided the patient into two groups according to their disease.

Income and productivity developments for all cases were averaged for points 1–4 and presented graphically.

### Procedure for evaluating the influence of Ada DX

To visualize the financial impact of an earlier diagnosis with the DDSS Ada DX, time point 2 (diagnosis by Ada DX) was included in the overall graph of income and productivity development. The number of patients for whom a worsening, improvement, or stagnation of income was observed at time point two was specified.

### Statistics

The data were analyzed using descriptive statistics. No significance tests were performed because generalization beyond our sample was not possible due to the heterogeneous patient population. Excel and SPSS were used for visualization and analysis.

## Results

Of the 93 patients in the preliminary studies by Ronicke et al. and T. Willmen et al., 71 patient cases with 26 different diseases were analyzed. 22 patient cases were excluded because it was not possible to generate new data in interviews. Reasons were death, lack of current contact information, or unwillingness to participate in interviews. In spite of the heterogeneity of the rare disease it was possible to divide them into two groups. The first one consisted of *n* = 28 patients suffering from symptoms of muscles and joints, and a second one of *n* = 43 with connective tissue diseases.

Table 1 shows an overview of the 71 patients (Table 1).

**Table 1:** Overview of the included patient cases (*n* = 71). The exchange rate for the US dollar is based on the reporting period (February 2023)

	Female			Male			Total		
	**N**	Joint, muscle, connective-tissue disorders	Vasculitides	**N**	Joint, muscle, connective-tissue disorders	Vasculitides	**N**	Joint, muscle, connective-tissue disorders	Vasculitides
**Number of included cases**	**46.00**	31.00	15.00	**25.00**	12.00	13.00	**71.00**	43.00	28.00
**Average age at interview**	**49.8**	51.29	46.73	**54.52**	54.08	54.92	**51.46 (48.2–54.7)**	52.69	50.83
**Average lost work time (months)**	**114.04 (0-361)**	105.33	150.6	**83.4 (0-217)**	76.30	97.6	**108.23 (0-361)**	90.82	124.1
Full-time	12.00	6.00	6.00	9.00	2.00	7.00	21.00	8.00	13.00
Part-time	8.00	4.00	4.00	0.00	0.00	0.00	8.00	4.00	4.00
Unemployed	8.00	7.00	1.00	3.00	2.00	1.00	11.00	9.00	2.00
Retired	18.00	14.00	4.00	12.00	8.00	4.00	30.00	22.00	8.00
Caregiver for kids	0.00	0.00	0.00	1.00	0.00	1.00	1.00	0.00	1.00
**Net income at interview (€)**	**1524.46**	1431.45	1716.67	**2450.00**	1885.42	2971.15	**1850.35**	1558.14	2299.11
							**(1585.20–2115.50)**	(1261.32–1854.96)	(1846.97–2751.24)
**Net income at interview (US-$)**	**1629.27**	1529.86	1834.69	**2618.44**	2015.04	3175.42	**1977.56**	1666.45	2454.04

### Development of income and productivity over the course of the disease

**Fig. 1:**
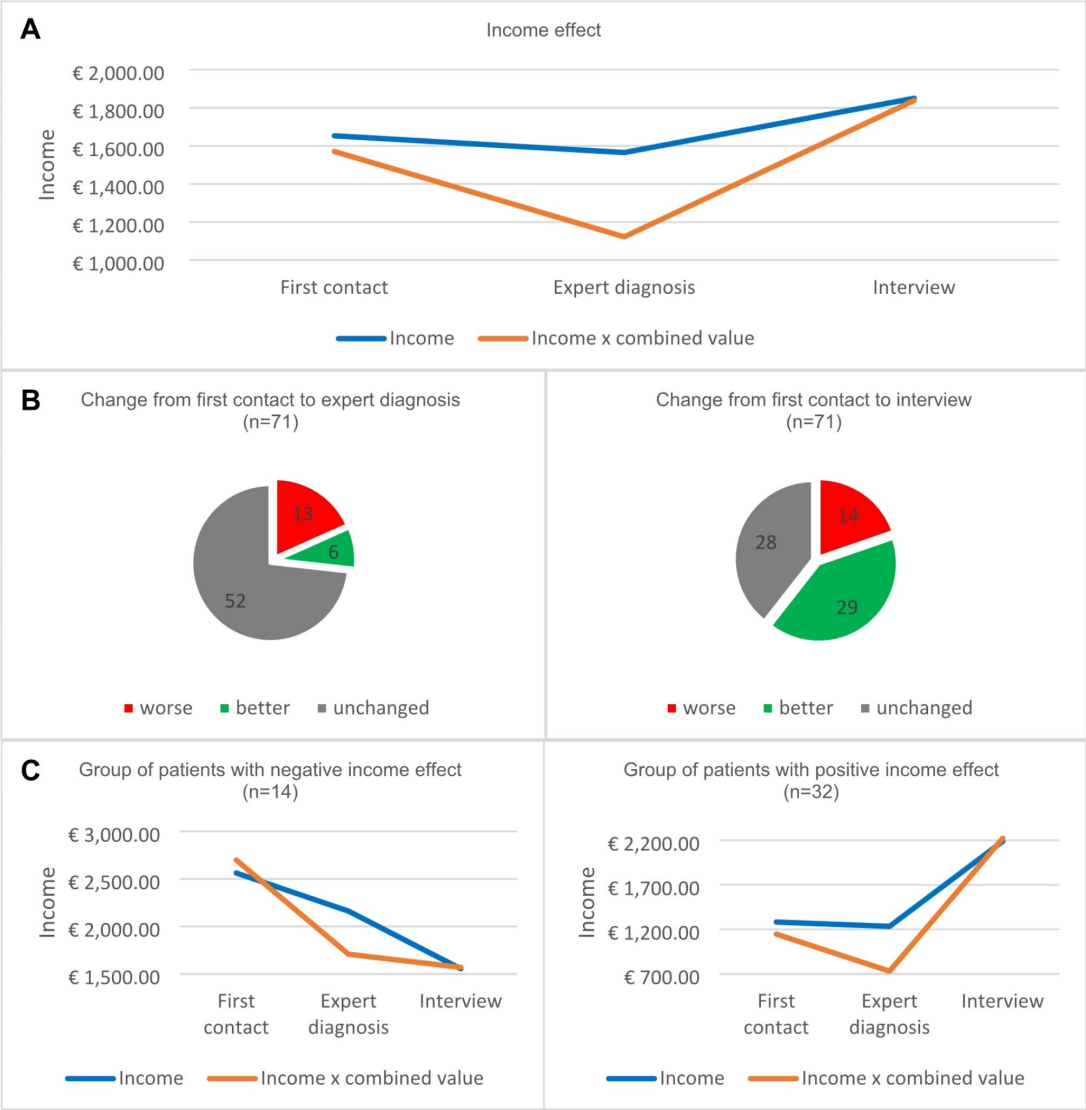
Income and productivity development at the time of the first contact, expert diagnosis and at the time of the interview. **A**: All income and productivity developments for all cases at time of the first contact, expert diagnosis and at the time of the interview. **B**: Illustrations of improvements, deteriorations and stagnations of incomes at time of expert diagnosis and at the time of the interview. **C**: Specific analysis of the groups with worsened or improved income over the course of the disease

Figure 1 shows the income and productivity development of the patient collective over the course of the disease. It can be seen that the income of 35.21% of the examined patients was constant during the study period. This was also reflected in the sum of the value for absenteeism and presenteeism, which remained unchanged.

A positive income development was observed in 43.66% of the patients from the onset of symptoms to the last interview time when the patients were in treatment with the correct diagnosis. Patients with a positive net income development had an average income of €1282.26 at the onset of the first symptoms. From this time until diagnosis, a slightly negative effect in income was observed (Fig. 1B). With the correct diagnosis, the net income of the patients increased to an average of €2189.52. The reason for the positive income development is a gain in productivity due to the correct diagnosis and start of therapy. If the combined value is considered, the combined income triples from the time of diagnosis to the time of interview.

With the onset of the disease, 19.72% of the patients experienced a loss of income, which did not change even with the correct diagnosis (Fig. 1C).

Analyzing the group of patients whose income had worsened since the onset of the first symptoms, it is noticeable that the correct diagnosis had no influence. The income decreased from an initial average value of €2562.50 to a final value of €1553.57 during therapy. In 8 of 14 patients, the deterioration of income went hand in hand with retirement. Here the income development correlates with the combined value, which decreased from the beginning of the disease to zero at the time of the interview.

### Effects of early retirement

At the time of the interview, 30 of 71 patients had already retired. Only one of the retired patients was still working until the statutory retirement age of 66.2 years.

The 29 patients in retirement had retired at an average age of 48.83 years. Nine patients had retired before the correct diagnosis and only 20 patients had retired with the correct diagnosis.

**Fig. 2:**
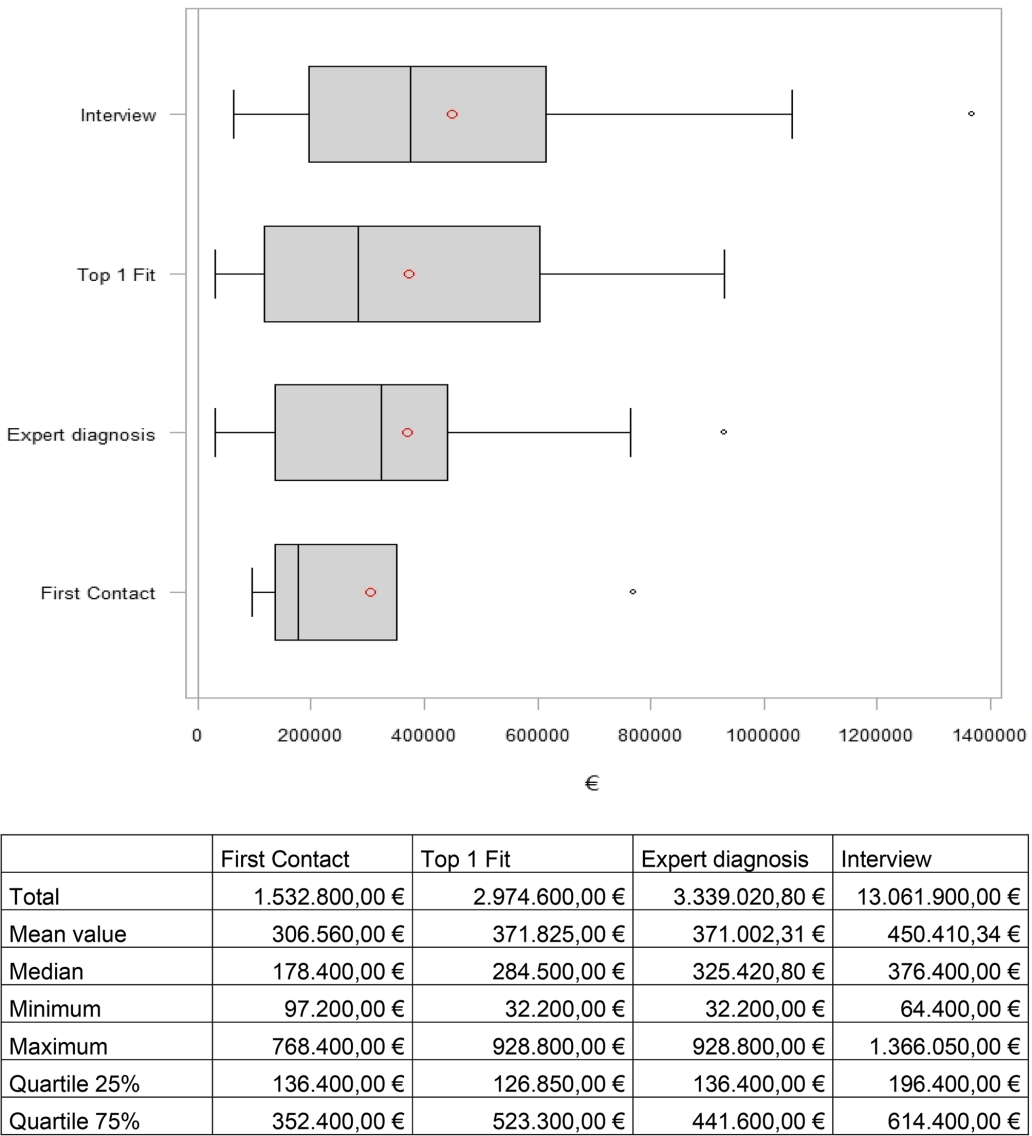
Loss of income of patients affected by early retirement. The black line represents the median. The red circle indicates the mean value. The black circle indicates the maximum.  Supplementary table to the box plot shown above

For twelve patients, retirement had no effect on net income. For nine patients, net income worsened as a result of retirement, and for nine patients, net income improved as a result of retirement. For patients who had a decrease in income, this was an average of €1041.67, and for patients who had an increase in income due to retirement, it was an average of €805.56. Figure 2 shows the income effect due to early retirement. The net loss up to the statutory retirement age for the patients who have retired to date is €13,061,900.00. Assuming that more of the remaining 41 patients retire early, the trend is upward.

### Income and productivity development depending on the type of disease

**Fig. 3:**
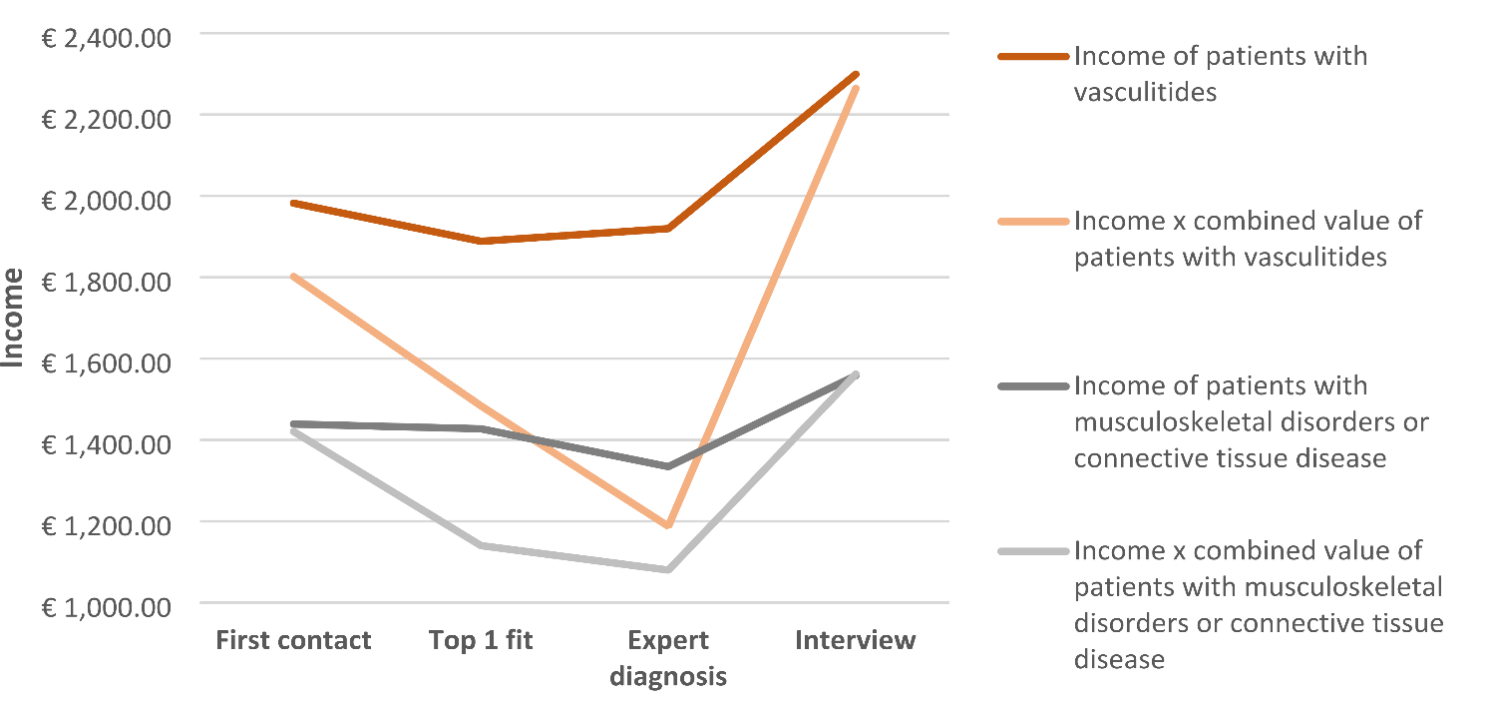
Comparison of income and productivity effects in patients with rare vascular diseases (n:28) and in patients with rare diseases of the musculoskeletal system or connective tissue (n:43) at the time of the first contact, expert diagnosis, top 1 fit of the DDSS and at the time of the interview

Figure 3 compares the impact on income and productivity in the course of rare vascular diseases (*n* = 28) with that of rare muscle, joint, and connective tissue diseases (*n* = 43).

When comparing rare small or large vessel vasculitides and rare diseases with joint, muscle and connective tissue involvement, the financial impact of those with joint, muscle and connective tissue involvement appears to be more significant (Fig. 3).

Patients with joint, muscle and connective tissue disease were more frequently affected by loss of income (23.26%) than patients with rare vasculitides (14.29%). While 50.00% of patients with vasculitides experienced an improvement in income as a result of the correct diagnosis and thus the correct therapy, this was the case for only 39.53% of patients with joint, muscle and connective tissue diseases.

Among patients who had a positive income development due to correct diagnosis, net income increased by an average of 210.40% for rare disease patients with joint, muscle and connective tissue involvement and by 154.70% for patients with rare small or large vessel vasculitides.

For patients with a constant negative income development, the average net income loss was €1052.50 for rare diseases with joint, muscle and connective tissue involvement and €875.00 for rare vasculitides. In that case income and productivity development correlate and run almost parallel to each other. It appears that the gradient in the representation of small or large vessel vasculitides is much steeper than the productivity representation of rare diseases with joint, muscle and connective tissue involvement, if as well productivity decreases with the onset of initial symptoms and rises with the onset of therapy. In general, it was found that the study participants with vasculitides had a higher income than those with joint, muscle and connective tissue diseases.

### Income and productivity development through Artificial Intelligence

**Fig. 4:**
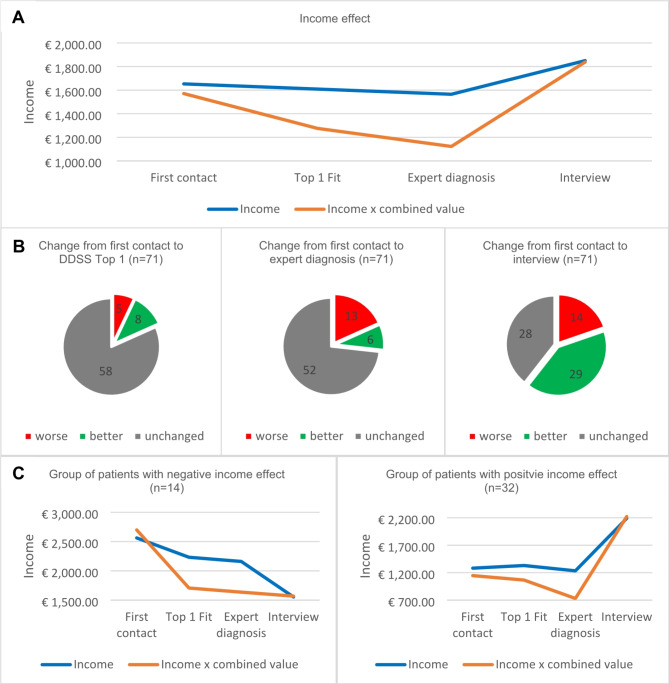
Income and productivity development at the time of the first contact, expert diagnosis, top 1 fit of the DDSS and at the time of the interview. **A**: Income and productivity effects for all cases at time of the first contact, expert diagnosis, top 1 fit of the DDSS and at the time of the interview. **B**: Illustration of improvements, deteriorations, and stagnations in income at time of top 1 fit of the DDSS, expert diagnosis, and at the time of the interview. **C**: Specific analysis of the groups with worsened or improved income with consideration of the DDSS over the course of the disease

Figure 4 presents the hypothetical impact on income and productivity that a DDSS could have produced. The use of a DDSS could have had a positive effect on income development as can be seen here (Fig. 4). For 27 of 71 patients the DDSS top 1 fit proposal would have resulted in an income improvement of €986.11 (net) per month on average, earlier than at the time of the interview.

For 10 patients whose income had decreased steadily from the onset of the disease to the condition under therapy, an earlier top 1 suggestion could have had a positive impact on a deterioration of €950.00 net per month on average.

Graph 4, which presents the income of all 71 patients at the time points when DDSS Ada DX would have indicated the correct diagnosis as a top 1 suggestion, shows the negative income development in the long diagnostic path of the patients. At.

top 1 the income loss is 2.66% and at the time of actual diagnosis, it is 5.32%.

## Discussion

Our study showed that the presence of a rare disease had a significant impact on working ability. Out of 30 retired patients, only one retired at the statutory retirement age, which is significantly higher than the German average [24]. Several other authors also confirm earlier retirement due to illness in the case of a rare disease [25–29]. Booth et al. reported that 40.45% of a patient cohort with systemic lupus erythematosus (SLE) retired early from working life for health reasons. Bjerrum et al. reported similar for patients with primary Sjögren’s syndrome. More than 50% of the patients who were employed at the beginning of the study gave up their jobs and received an early retirement or disability pension [29].

Our study made visible that over the very long diagnostic odysseys of up to 28.75 years, no changes in income were quantifiable in 35% of the patients. This could indicate lower career advancement opportunities, which is also reflected in a European survey of patients with rare diseases where 67% of the respondents reported lower career advancement opportunities due to their disease [14].

Our results also show the correlation of salary and productivity. The correct diagnosis combined with the correct treatment could lead to an improvement in income for a large proportion of patients, as their productivity increased after starting treatment. The importance of access to proper treatment in terms of indirect health care costs was shown by Andreu et al. While patients with rare diseases, who could be treated, incurred annual indirect costs of $40,000, the indirect costs for patients without treatment were $73,000 [12].

Thus, our hypothesis that a correct diagnosis has a positive effect on patient income was confirmed in at least 38% of the patient cases.

Our study could also prove that the economic effects for patients with joint, muscle and connective tissue disease are more severe than for those with small or large vessel vasculitis, which makes it possible to confirm another hypothesis. This hypothesis has been confirmed in other studies as well. Some authors describe that about a quarter of patients with rare vasculitides are unable to work [30, 31], whereas patients with rare diseases with joint, muscle and connective tissue involvement are significantly more often unable to work mainly due to fatigue [28, 29].

To date, there have been no studies on the impact of earlier diagnosis by a DDSS on income development in patients with rare diseases. Only very few studies address the economic efficiency of a DDSS in the diagnostic process [7, 18]. However, the results of this study show the potential of a DDSS to optimize the diagnostic process and thus positively influence income development.

### Limitations

The study was intended to represent as broad a spectrum of rare diseases as possible by dividing them into two subgroups; thus, the results of the study cannot be broken down further into smaller groups of patients. The small study sample and the unknown population of the patients did not permit to apply robust statistical tests, and also non-parametric procedures could not be applied.

Furthermore, there is no control group due to the rarity and heterogeneity of the studied diseases.

Internationally, the impact of rare diseases on income can be very different, as retirement systems and retirement ages vary widely.

Due to the retrospective study design, the impact of an earlier diagnosis by a DDSS on patients’ income cannot be proven and the results only suggest that if a DDSS had suggested the correct diagnosis earlier, this would also have led to financial benefits for the patients.

### Outlook

While the economic impact of many of the analyzed patient cases was relatively low, as deficits were offset by disability insurance or earlier retirement, the fiscal impact of early retirement and reduction in earning capacity are likely to be significant. It would be of interest to examine the fiscal impact of rare diseases on the patient population. A study by Connolly et al. has already highlighted the fiscal impact of rare diseases using the example of hereditary transthyretin-mediated amyloidosis (hATTR) [32].

It would also be interesting to calculate the amount of “hidden” costs, e.g. the loss of work compensated for through the support of relatives.

The long diagnostic odysseys for patients with rare diseases, the health-related constraints and by implication, the financial burdens, are a limitation to the quality of life. For further studies, it would be interesting to see to what extent the correct diagnosis can influence the quality of life of the investigated patients.

## Conclusion

In conclusion, rare diseases strongly influence the economic situation of patients with rare diseases. After the onset of the first symptoms, 18.31% of the patients experienced a decrease in income. Only 8.45% of patients were able to improve their income between the onset of the first symptoms and diagnosis, while 73.24% of the patient experienced a stagnation in income.

After the diagnosis was made, 38.03% of the patients experienced an improvement in income, illustrating the importance of diagnosis on the economic situation. For a quarter of these patients, a pension could be obtained through the correct diagnosis, and the remaining three quarters were able to return to their former or better work performance upon correct therapy.

The impact on patients and the economic effects depend on the type of disease. Our study showed that patients with a rare disease with joint, muscle and connective tissue involvement were more frequently affected by income loss. The amount of the loss of income was also higher for patients with a rare disease with joint, muscle and connective tissue involvement.

The results of the study suggest that the use of DDSS Ada DX would have the potential to reduce the patient’s income loss. Ada DX would have already allowed for the correct diagnosis with an average monthly net income loss of 2.66%.

## Data Availability

The data analyzed in this study are included in this published article. The data supporting the findings of this study are available upon request from the corresponding author ADW. The data are not publicly available because they contain information that could compromise the privacy and/or consent of study participants.

## Abbreviations

AI: Artificial Intelligence
DDSS: Diagnostic Decision Support System
WHO: World Health Organization
HPQ: Health and Performance Questionnaire
MHH: Hanover Medical School
hATTR: hereditary transthyretin-mediated amyloidosis
